# Meniscus Work and Implant Selection Are Major Cost Drivers of Anterior Cruciate Ligament Reconstruction

**DOI:** 10.7759/cureus.34647

**Published:** 2023-02-05

**Authors:** Tommy Pan, Jacob Gottshall, Tonya S King, Robert A Gallo

**Affiliations:** 1 Orthopedic Surgery, Penn State College of Medicine, Hershey, USA; 2 Internal Medicine, Penn State College of Medicine, Hershey, USA; 3 Public Health Sciences, Penn State College of Medicine, Hershey, USA; 4 Orthopedics and Rehabilitation, Penn State Health Milton S. Hershey Medical Center, Hershey, USA

**Keywords:** allograft, autograft, meniscus repair, partial meniscectomy, financial analysis, anterior cruciate ligament reconstruction

## Abstract

Background

The current study examines the financial charges associated with primary anterior cruciate ligament reconstruction (ACLR), specifically the contribution of graft choice, graft type, and concomitant meniscus surgery, in the outpatient hospital setting.

Methods

A retrospective financial billing review was performed on patients who underwent ACLR at a single academic medical center from January to December 2019. Age, BMI, insurance, length of operation, regional block, implants, meniscus surgery, graft type, and graft choice were extracted from hospital electronic patient records. Charges attributed with graft, anesthesia services, supplies, implants, surgeon fees, radiology charges, and total charges were collected. Total amount that insurance and patient paid were also obtained. Descriptive and quantitative statistics were performed.

Results

A total of 28 patients were studied (18 males, 10 females). The average age was 23.8 years. There were 20 concomitant meniscus surgeries. Six allografts and 22 autografts were used [eight bone-patellar tendon-bone (BPTB), eight hamstrings, six quadriceps]. The average and median total charge was $61,004 and $60,390, respectively (range: $31,403 to $97,914). The average insurance paid was $26,045 while out-of-pocket costs were $402. The average paid by private insurance was higher compared to government insurance ($31,111 vs. $11,066, p<0.001). Graft choice such as allograft vs. autograft (p=0.035) and meniscus surgery (p=0.048) were significant factors to the overall cost.

Conclusions

Graft choice, specifically the quadrupled hamstring autograft, and concomitant meniscal surgery are major contributors to variations in ACLR charges. Decreasing implant and graft costs and limiting surgical time can decrease charges associated with ACLR. We hope these findings can help guide surgeon financial decisions, by demonstrating the need to take into account the increased total charges and amount paid associated with specific grafts, meniscus surgery, and prolonged OR time.

## Introduction

The anterior cruciate ligament (ACL) is one of the most commonly injured ligaments requiring surgery in the United States [1-4]. Of the approximately 250,000 annual cases of ACL tears in the United States, at least 100,000 of those cases are reconstructed [5]. Anterior cruciate ligament reconstruction (ACLR) has been proven to be an effective procedure for alleviating knee instability and expediting return to sporting activity in active populations, and has been associated with excellent reported outcomes in greater than 90% of patients [6-9]. A growing incidence in ACLR has also been observed over the past decade, particularly in females and patients younger than 20 or older than 40 years of age [10]. ACLR has become a high-volume procedure in the United States: 95% of procedures occurred as outpatient surgeries in 2006, a sharp rise compared to 43% in 1994 [7, 11]. This trend in ACLR demonstrates a need to consider its cost, especially as the US healthcare system transitions toward value-based care [12]. Value-based care is a form of reimbursement that ties payments for care delivery to the quality of care provided and rewards providers for efficiency and effectiveness. The goal is to provide better care for individuals, improve population health management strategies and reduce healthcare costs. Reimbursement is generally via “bundled” payments in which patients pay a single price covering the entire episode of care [11, 12].

Currently, the cost of ACLR is estimated to be $11,900, contributing to over $1 billion of the United States' annual healthcare cost [4, 13]. ACLR has been demonstrated to be cost-effective [14]. Multiple studies have found that ACLR incurred significantly lower mean lifetime cost to society per patient, in both the intermediate ($27,452 versus $32,276) and long term ($38,411 versus $92,786) compared to nonoperative treatment [15]. Furthermore, ACLR has also been found to increase the quality-adjusted life years (QALY), having a cost of $20,612 per QALY compared to $23,391 per QALY in nonoperative treatment, with a cost-effective ratio of $4890/QALY [5]. However, to our knowledge, few studies investigate the financial impact of concomitant meniscus surgery in ACLR.

The current study examines the financial charges associated with ACLR in the outpatient hospital setting. We hypothesized that graft choice, graft type, and concomitant meniscus surgery significantly contribute to the charges associated with ACLR. We hope our findings can guide surgeons' financial decision-making by shedding light on factors associated with increased total charges and amount paid in ACLR.

## Materials and methods

This level II evidence study was approved by the College of Medicine Institutional Review Board. Retrospective chart and financial billing reviews were performed on 28 consecutive patients who underwent primary ACLR from January 2019 to December 2019 at a single academic institution. Exclusion criteria were: multiligamentous knee injury and age < 18. Three fellowship-trained sports medicine surgeons operated on the patients.

Data extracted included: age, gender, ethnicity, body mass index (BMI), surgeon, length of operation (LOO), regional block used, implants used, associated meniscus surgery in addition to ACLR (yes or no) and if so, what type of meniscus surgery (partial meniscectomy or repair), graft type [allograft (ALLO) versus autograft (AUTO)], and autograft choice, including bone-patellar tendon-bone (BPTB), quadrupled hamstring (HAM) or quadriceps tendon (QUAD).

Financial information extracted included charges associated with grafts, anesthesia, radiology, pharmacy, implants, supplies, operating room (OR), anesthesiologist, and surgeon. The total charges and final amount that insurance and patient paid were also obtained. LOO was defined as incision start time to surgery end time. Surgical stage reflected the OR charge. Shared charges reflected the surgical fee from the hospital based on OR charge. Individual surgeons’ and anesthesiologists’ professional fees were billed separately. We define “cost” as the exact dollar amount the hospital was compensated to cover the ancillary and direct operating room charges of ACLR within the 90-day care window. Charges for intraoperative imaging were included under radiology and charges for durable medical equipment were included under supplies. Primary insurance type was also extracted and subcategorized as government or private. The charges that the insurance pays depend on the type of insurance taken by the patient as patients can take a better insurance for a higher cost cover.

Descriptive statistics were used, including frequencies and percentages for categorical measures, and means and ranges for continuous measures. The distribution of each surgical outcome was evaluated for approximate normality and transformed using the natural log transformation, if necessary. Potential predictors were evaluated separately for each of the surgery outcomes using analysis of variance (ANOVA) and with simultaneous adjustment for other significant predictors using analysis of covariance (ANCOVA). Potential predictors that were compared included surgeon, LOO, graft choice, concomitant meniscus surgery, use of regional block, radiology, and insurance type. Results were reported in terms of model-adjusted means and 95% confidence intervals. Significance was defined as p<0.05, and statistical tests were performed using SAS statistical software version 9.4 (SAS Institute, Inc., Cary, NC).

## Results

Twenty-eight patients were studied (18 males, 10 females). The average age was 23.8 years (range: 14 to 55 years) and BMI was 25.8 (range: 21 to 40). Ethnicities included Caucasian (21), Hispanic (5), African American (1), and Asian (1). All procedures were performed in the outpatient setting. The procedures were split among Surgeon 1 (4), Surgeon 2 (17), and Surgeon 3 (7). The average LOO was 115.5 min (range: 58 to 174 min). Regional adductor nerve block was used in seven patients and radiology was used in 12 patients. Duration of follow-up averaged 9.8 months (range: 3 to 24). No known complications occurred during the 90-day recovery period. Financial considerations of postoperative complications were not modeled in the analysis.

There were 20 concomitant meniscus surgeries (five partial meniscectomies, 15 repairs). There was no instance where both meniscus repair and meniscectomy were performed. Billing was the same regardless if both medial and lateral meniscus work were performed vs. medial or lateral only. Six allografts and 22 autografts were used, including BPTB (8), HAM (8), and QUAD (6). Allograft type included pre-prepared BPTB (6).

The average and median total charges were $61,004 and $60,390, respectively (range: $31,403 to $97,914). The average total paid was $26,447 (insurance paid $26,045 vs. patient paid $402).** **Table 1 illustrates the average categorical charge breakdowns associated with ACLR.

**Table 1:** Average Categorical Charge Breakdown Associated with ACLR ACLR: Anterior cruciate ligament reconstruction; OR: Operating room.

Category Type	Average Charge (%)
ACLR Procedure Fee	$27,455 (45.0%)
Anesthesia	$4064 (6.7%)
Anesthesiologist Fee	$2410 (3.9%)
Total Supply	$14,202 (23.3%)
Implant	$5018 (8.2%)
OR Fee	$4613 (7.6%)
Pharmacy	$693 (1.1%)
Physical/Occupational Therapy	$830 (1.4%)
Radiology	$232 (0.4%)
Surgeon Fee	$7332 (12.0%)
Other	$1527 (2.5%)

Table 2 illustrates examples of the common implant and supply charges associated with ACLR.

**Table 2:** Common Individual Implant and Supply Charges in ACLR ACLR: Anterior cruciate ligament reconstruction

Supply and Implant Type	Charges ($)
Surgical Suction Mat	2725
Kit EndoButton	2720
Fast-Fix 360 Meniscus Repair (Smith & Nephew)	2559
ArthroCare ArthroWand	2233
Bioabsorbable Sheath Biointrafix Flexisheath Large (DePuy Mitek Inc)	1721
EndoButton CL	1602
Tibial Screw Bioabsorbable (DePuy Mitek Inc)	1565
Interference Screw (Smith & Nephew)	1510
Probe	1196
Surgical Electrode	766
Pusher Fix Knot	740
Flexible Pin	735
Knee Arthroscopy Set	347
Arthroscopy Tubing	277
Plastic Cannula Plug	213

Graft choice, use of regional block, meniscus surgery, and radiology were all significant predictors of total charges when evaluated separately; however, when evaluated simultaneously in the multivariable model, graft choice and meniscus surgery were the only significant predictors for total charges. The cost breakdown of the graft choice and types are illustrated in Table 3. There was a significant difference in average total charge among the graft choices (p=0.035 with adjustment for meniscus surgery type), driven by the comparisons between HAM versus BPTB (p=0.026) and HAM versus QUAD (p=0.030).

**Table 3:** Cost Breakdown of the Graft Allograft Versus Autograft and Graft Choices BPTB: Bone-patellar tendon-bone

Graft Type	Cases	Average Total Charge ($)	Average Total Paid ($)	Average Implant Charge ($)	Average Length of Operation (min)
Allograft BPTB	6	64,864	11,316	3033	100.7
Autograft	22				
BPTB	8	55,824	23,811	1806	127.5
HAM	8	71,466	33,262	3731	113.5
QUAD	6	53,999	15,964	2799	117.0

Graft choice and insurance type were both significant predictors of total paid. The overall difference in average total paid among the three graft choices (p=0.022, with adjustment for insurance type) was driven by the comparison between HAM and QUAD (p=0.006). The average total paid for ALLO was also significantly lower than BPTB (p=0.035) and HAM (p=0.001). However, the amount paid also appeared related to the payer (private vs. government insurance), as the government reimbursed $11,000 compared with over $30,000 for private insurance. 3/6 (50%) of ALLO patients had private insurance. Thus, even though this payment data was adjusted for insurance type, a larger sample size confirming if the majority of government-insured patients were treated with ALLO may reveal if the effect on the amount paid is due to insurance rather than graft choice itself.

The distribution of implant charge was highly skewed, and improved with natural log transformation. Therefore, means were estimated on the natural log scale, exponentiated, and reported as geometric means. Graft choice and surgeon were significant predictors of implant charge. The difference in implant charge among the three graft choices (p=0.025 with adjustment for surgeon) was driven by the comparison between BPTB and HAM ($1806 vs. $3731, respectively, p=0.004). However, the surgeon who performed the most HAM reconstructions also performed the most meniscus repairs using the Fast-Fix device, which was the second most expensive device listed in Table 2**. **The cost difference between BTB and HAM was just as likely, if not more, to be driven by the use of the all-inside meniscus repair device. There was not a difference in average LOO among graft type or choice (p=0.27).

There were eight government (Medicare or Medicaid) and 20 private insurance types. The average total charge by government insurance was higher compared to private insurance ($63,256 vs. $60,103). There was not a significant difference in the average total charges between insurance types (p=0.66). However, the average total paid by private insurance was higher compared to government insurance ($31,111 vs. $11,066, p<0.001), adjusting for graft type and choice.

The cost breakdown by meniscus surgery is illustrated in Table 4**.** The overall comparison of average total charge among the three meniscus surgery types was statistically significant (p=0.048 with adjustment for graft type/choice), driven by the comparison between partial meniscectomy and no meniscus surgery ($71,908 vs. $50,066, respectively, p=0.018). There was no significant difference in average total paid among the types of meniscus surgery (p=0.55), but there was a difference in average LOO among the types (p=0.015). Partial meniscectomy had significantly shorter LOO than no meniscus surgery (p=0.041) and repair (p=0.004).

**Table 4:** Cost Breakdown by Meniscus Surgery

Type of Meniscus Surgery	Cases	Average Total Charge ($)	Average Total Paid ($)	Average Length of Operation (min)
Meniscus Repair	15	62,641	28,887	124.47
Partial Meniscectomy	5	71,908	18,806	88.4
None	8	50,066	26,651	115.62

The surgeon cost breakdown is illustrated in Table 5**.** Surgeon 1 primarily performed meniscectomies (75%), Surgeon 2 primarily performed repairs (71%), and Surgeon 3 performed 14% of meniscectomies and 29% of repairs (p=0.013). The average implant charge was significantly lower for Surgeon 3 ($990) compared to Surgeon 1 ($4025, p=0.012) and Surgeon 2 ($5219, p=0.004). After adjusting for meniscus surgery, there was no difference in average implant cost among surgeons (p=0.11). With regards to graft choice, Surgeon 1 primarily used ALLO, Surgeon 2 primarily used BPTB and HAM, and Surgeon 3 primarily used QUAD.

**Table 5:** Cost Breakdown by Surgeon

Surgeon	Cases	Graft Type/Choice (ALLO/BPTB/HAM/QUAD%)	Average Implant Charge ($)	Average Total Charge ($)	Average Length of Operation (min)
1	4	75/25/0/0	4025	70,627	81.5
2	17	12/41/47/0	5219	62,405	120.5
3	7	14/0/0/86	990	52,101	122.7

## Discussion

The current study sheds light on and investigates the rate and impact that concomitant meniscus surgery has on the overall costs associated with ACLR in the outpatient setting. Approximately 55-65% of ACL injuries have an associated meniscus tear, treated with partial meniscectomy, debridement, and/or repair [16, 17]. Meniscus repair typically incurs higher costs compared to meniscectomy; however, repairs are more cost-effective in the long term due to decreased risk of osteoarthritis [7, 8, 18]. Nevertheless, meniscectomy is performed 2-3 times more frequently compared to repairs in ACLR [18-20].

In 1996, Novak et al. demonstrated a greater than $7000 per case reduction in ACLR charges in the outpatient vs. inpatient setting [4]. With the majority of ACLR in the United States performed in the outpatient setting, our focus shifted towards comparing meniscus repair, meniscectomy, and no meniscus treatment due to the different complexities between procedures. We highlighted the variance of overall charges between individual surgeons in their preference for the type of meniscus surgery, graft choice, and implants used.

In the current study, meniscus repairs outnumbered meniscectomies by 3 to 1. Investigating the implants associated with meniscus repairs, the Fast-Fix suturing devices used in the all-inside technique incurred the highest charge. There were four cases involving greater than three Fast-Fix devices used, all in Surgeon 2, supporting the higher overall implant charges compared to Surgeon 1, who mostly performed meniscectomies. After controlling for meniscus surgery, there was no difference in average implant charges in ACLR. Therefore, we recommend further studies comparing the charges associated with all-inside devices versus inside-out repair in ACLR to shed more light on the most cost-effective meniscus surgery.

Many different techniques for grafting exist, including the bone-patellar tendon-bone (BPTB), quadriceps (QUAD), tibialis anterior (TA), and quadrupled hamstring (HAM) tendon autograft and allografts [12-15]. The BPTB autograft is a popular graft choice due to its proven long-term efficacy and low failure rates [12]. However, the quadrupled hamstring has risen in popularity and is the most commonly used graft across the world [13]. HAM has demonstrated comparable outcomes and incurs less time, surgical cost, and hospital charges compared to BPTB [21]. In the current study, BPTB and HAM autografts were most commonly used. HAM incurred a significantly higher total charge compared to BPTB and QUAD, with QUAD being substantially less expensive than HAM. Part of this difference could be attributed to 5 of 8 (62.5%) ACLR involving HAM that also had a concomitant meniscus repair, which accrued longer operating time and greater implant costs. Furthermore, Surgeon 2 performed mostly all-inside meniscus repairs as opposed to an inside-out technique. As a result, the average implant charge of HAM was more than double of BPTB and 33% higher than QUAD. A larger sample size comparing HAM with other autografts while controlling for meniscus repair can better determine the association.

An estimated 20-25% of ACLR are performed using allografts, which are found to be more cost-effective compared to autografts by reducing OR time, overhead costs, operative complications, and recovery time [13, 15]. In the current study, six (21%) ACLR involved pre-prepared BPTB allografts; however, we did not find any difference in total charge between graft types.

We also highlighted the impact insurance type can have on the total amount paid in ACLR: there was a significant difference in the amount paid between government and private insurance payers. Hospital or payer contracts may explain this large discrepancy because contract pricing ultimately determines reimbursement and impacts healthcare spending. We recommend further cost analysis studies with larger sample sizes to determine differences in payments between patients with government and private insurance.

It is important to consider that surgeons should not choose grafts and implants strictly by looking at the cost. For example, the quadrupled hamstring graft is widely considered to be a superior graft despite having a higher cost when compared to BPTB graft in primary ACLR. On the other hand, BPTB has been shown to be a better graft in revision ACLR. Rather, the current study highlights that conscious efforts should be made to streamline implant costs and make them more affordable in ACLR.

With the recent financial pressures placed on the healthcare system combined with the emphasis on high-value care, orthopedic surgeons now have to justify their procedures based on a time, cost, and outcome analysis [16, 17]. Although ACLR is one of the most widely performed orthopedic surgeries, few studies have examined the financial impact of meniscus surgery in the overall charges associated with ACLR. The current study sheds light on this topic while also exploring other potential factors. We recommend further studies with more surgeons investigating the total charges and amount paid for meniscus surgery in ACLR in the outpatient setting to better understand the overall financial picture.

Limitations of the current study include: 1) small sample size, 2) all procedures performed in the outpatient setting at an academic hospital instead of a surgery center, 3) disparity of private versus governmental payers, and 4) potential confounding effect of using predominantly the all-inside meniscus repair device rather than inside-out suture repair. The relatively small sample size and use of an academic hospital were result of data access and availability. Due to the limited sample size and all ACLR performed in the hospital setting, our results may not be representative of the standard of care across the world. The majority of ACLR in the United States are performed in ambulatory surgery centers, many are physician-owned. In these centers, particularly those physician-owned, it is possible that costs are more closely monitored and decisions on implants, repair techniques, and “extras”, such as suction mats and radiofrequency ablation wands, are made with more concern for cost. For example, the costs of all-inside meniscus repair implants were a major cost driver in this study but may be substituted for inside-out sutures, which would likely decrease implant costs but may increase surgical times. Despite these limitations, this study does provide insight into some of the variables associated with increased charges for ACLR.

## Conclusions

In this study, we demonstrate that graft choice, specifically the quadrupled hamstring autograft, and concomitant meniscal surgery are major contributors to variations in ACLR charges in the outpatient setting. We hope these findings can help guide surgeons' financial decisions, by demonstrating the need to take into account the increased total charges and amount paid associated with specific grafts, meniscus surgery, and prolonged OR time. Ultimately, conscious efforts should be made to streamline implant costs and make them more affordable in ACLR.
